# Drug therapy and catheter ablation for management of arrhythmias in continuous flow left ventricular assist device’s patients: a Clinical Consensus Statement of the European Heart Rhythm Association and the Heart Failure Association of the ESC

**DOI:** 10.1093/europace/euae272

**Published:** 2024-10-31

**Authors:** Petr Peichl, Antoni Bayes-Genis, Thomas Deneke, Ovidiu Chioncel, Marta deRiva, Maria Generosa Crespo-Leiro, Antonio Frontera, Finn Gustafsson, Raphaël P Martins, Matteo Pagnesi, Philippe Maury, Mark C Petrie, Frederic Sacher, Offer Amir

**Affiliations:** Department of Cardiology, IKEM, Vídeňská 1958/9, Prague, Czech Republic; Heart Institute at Hospital Universitari Germans Trias i Pujol, CIBERCV, Badalona, Spain; Clinic for Arrhythmology, Klinikum Nuernberg Süd, University Hospital of the Paracelsus Medical University, Nuernberg, Germany; Institute of Emergency for Cardiovascular Diseases, ‘C.C.Iliescu’ Bucharest, Romania; University of Medicine Carol Davila, Bucharest, Romania; Leiden University Medical Center, Leiden, The Netherlands; Complexo Hospitalario Universitario A Coruña (CHUAC)-CIBERCV, Instituto de Investigación Biomedica A Coruña (INIBIC), Universidad de A Coruña (UDC), A Coruña, Spain; Department of Cardiac Electrophysiology, Great Metropolitan Hospital Niguarda, Milan, Italy; Department of Cardiology, Rigshospitalet, Denmark; Department of Cardiology, University Hospital of Rennes, Rennes, France; Department of Medical and Surgical Specialties, Radiological sciences and Public Health, Institute of Cardiology, ASST Spedali Civili, University of Brescia, Brescia, Italy; Department of Cardiology, Rangueil Hospital of Toulouse, Toulouse, France; School of Cardiovascular and Medical Sciences, University of Glasgow, Glasgow, UK; Cardiac Arrhythmia Department, Univ. Bordeaux, CHU de Bordeaux, INSERM, CRCTB, U 1045, IHU Liryc, Bordeaux, France; Heart Center, Hadassah Medical Center and Hebrew University, Jerusalem, Israel

**Keywords:** Left ventricular assist device, Atrial fibrillation, Ventricular arrhythmia, Catheter ablation, Heart failure

## Abstract

Left ventricular assist devices (LVADs) are an increasingly used strategy for the management of patients with advanced heart failure. Although these devices effectively improve survival, atrial and ventricular arrhythmias are common with a prevalence of 20–50% at one year after LVAD implantation. Arrhythmias predispose these patients to additional risk and are associated with considerable morbidity from recurrent implantable cardioverter-defibrillator shocks, progressive failure of the unsupported right ventricle, and herald an increased risk of mortality. Management of patients with arrhythmias and LVAD differs in many aspects from the general population heart failure patients. These include ruling out the reversible causes of arrhythmias that in LVAD patients may include mechanical irritation from the inflow cannula and suction events. For patients with symptomatic arrhythmias refractory to medical treatment, catheter ablation might be relevant. There are specific technical and procedural challenges perceived to be unique to LVAD-related ventricular tachycardia (VT) ablation such as vascular and LV access, signal filtering, catheter manoeuvrability within decompressed chambers, and electroanatomic mapping system interference. In some patients, the arrhythmogenic substrate might not be readily accessible by catheter ablation after LVAD implantation. In this regard, the peri-implantation period offers a unique opportunity to surgically address arrhythmogenic substrate and suppress future VT recurrences. This document aims to address specific aspects of the management of arrhythmias in LVAD patients focusing on anti-arrhythmic drug therapy and ablations.

## Table of contents

Introduction
Aim of the paper
General considerations
Drug therapy for arrhythmias in LVAD patients
 HF guideline-directed medical therapy
 AAD therapy
 Atrial arrhythmias
 Ventricular Arrhythmias
 Amiodarone use in post-LVAD arrhythmias
Catheter ablation in LVAD recipients
 Catheter ablation of atrial arrhythmias
 Catheter ablation of VAs prior to LVAD implantation
 Peri-implantation surgical VAs ablation
 Catheter ablation of VAs after LVAD implantation
 Management of ES–the role of drug therapy and catheter ablation
Procedural aspects of catheter ablation in LVAD patients
 Pre- and peri-procedural imaging
 Signal filtering and the role of ECG in the location of the VT exit site
 EMI and selection of mapping system
 Mapping strategies and ablation targets
 Complications of catheter ablation in LVAD patients
 Vascular cannulation and access to cardiac chambers
 Peri-procedural anticoagulation management
 Peri-procedural haemodynamic monitoring
Gaps in knowledge
Conclusions
Acknowledgements
References

## Introduction

Left ventricular assist devices (LVADs) are an increasingly used strategy for the management of patients with advanced heart failure (HF), defined as the persistence of severe symptoms despite the use of optimized medical, surgical, and device therapies.^1^ Although these devices effectively improve survival, atrial and ventricular arrhythmias (VAs) are common with a prevalence of 20–50% at one year after LVAD implantation.^2–5^ Arrhythmias predispose these patients to additional risk and are associated with considerable morbidity from recurrent implantable cardioverter-defibrillator (ICD) shocks, progressive failure of the unsupported right ventricle, and herald an increased risk of mortality.^6^

## Aim of the paper

Management of patients with VAs and LVAD differs from the general population of non-LVAD HF patients. Current guidelines of the European Society of Cardiology^7^ mention the LVAD patient population very briefly (one recommendation regarding the ICD implantation indication). Guidelines do not provide clinical and practical advice on anti-arrhythmic drug (AAD) management and specific aspects of catheter ablation and peri-implantation arrhythmia intervention for LVAD patients nor is there a clinical reference for managing LVAD patients in acute arrhythmic setting [such as electrical storm (ES)]. This document aims to expand on the current guidelines.

The writing group has been comprised of cardiovascular specialists, each having extensive experience with cardiac arrhythmias and LVADs. As the management requires a multidisciplinary approach, this *clinical consensus statement* represents the consensus of a panel of experts from the European Heart Rhythm Association (EHRA) and Heart Failure Association (HFA).

**Figure 1:**
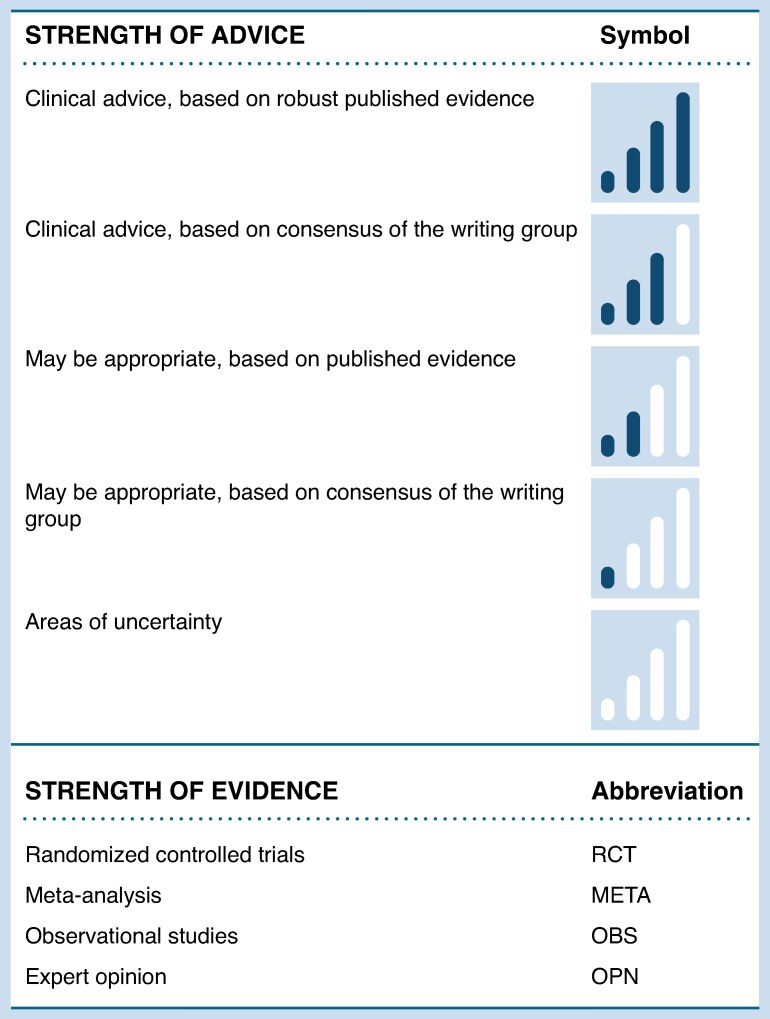
Table of advice structure.

The lack of randomized trials makes the guidelines level recommendation a real challenge. This joint effort of HFA and EHRA provides a *clinical consensus statement* that aims to review available data, summarize expert opinion to guide the best medical practice (*Figure 1*) and identify the gaps in knowledge. Controversial issues regarding the management of arrhythmias in this specific population are discussed and advice for frequently encountered situations in clinical practice is provided.

The current document is focused on drug management and specific aspects of catheter ablation in LVAD recipients. Issues regarding cardiac implantable electronic devices (CIEDs) implantation, programming and deactivation during the ‘LVAD patient journey’ are covered in the complementary *clinical consensus statement* of the EHRA and the HFA of the ESC that are being published in the European Journal of Heart Failure^8^ (*Amir O. et al, EJHF, in press*).

## General considerations

Continuous-flow LVADs have become a standard of care in end-stage HF patients with the nowadays dominance of HeartMate3, a fully magnetically levitated centrifugal-flow pathway pump with a frictionless rotor and a fixed intrinsic pulse.^9^

Before LVAD implantation, atrial arrhythmias are diagnosed in 21% to 54% of patients, with the majority having atrial fibrillation (AF) and a minority experiencing atrial flutter and atrial tachycardia.^10^ While pre-implantation AF remains the most robust predictor of post-LVAD AF, ∼20% to 30% of patients will experience the onset of *de novo* AF following LVAD implantation.^4^ Following LVAD implantation, AF was found to resolve in 43% of patients with a paroxysmal form of the arrhythmia, a result likely associated with favourable remodelling in left atrial size and volume.^11^ Patients with LVAD and AF may face an increased risk of developing post-LVAD VAs.^5^

VAs are also prevalent following LVAD implantation. They occur with an incidence between 20% and 50% at one year.^3,5,12^ The high incidence of VAs in patients with LVAD is attributed to two main factors: mechanical causes related to the assist device itself and its insertion site, and causes arising from the underlying myocardial substrate.^13^ The pre-dominant VA in these patients is monomorphic ventricular tachycardia (VT), pointing to scar-related reentry as the primary mechanism. These reentrant VTs can originate either from the pre-existing fibrotic substrate related to underlying myocardial disease or from the formation of new apical scar due to the insertion of the inflow cannula.^14^ Several other mechanisms including high levels of sympathetic nerve activity and endothelial dysfunction were also suggested as a possible link between LVAD implantation and arrhythmias.^15^

Long-lasting VAs occur in the presence of the required haemodynamic support provided by the LVAD. While initially well-tolerated, extended periods of VA may contribute to increased mortality in LVAD patients.^16^ The context of arrhythmia treatment in general should include its impact on haemodynamic stability, its potential effect on the LVAD performance and if possible on the potential duration of the LVAD long-term plan (bridge vs. destination therapy).

## Drug therapy for arrhythmias in LVAD patients

### HF guideline-directed medical therapy

Guideline-directed medical therapy (GDMT) with angiotensin-converting enzyme inhibitors or angiotensin receptor-neprilysin inhibitors, beta-blockers (BB), MRA and sodium-glucose cotransporter-2 inhibitors has been shown to improve survival, reduce the risk of HF hospitalizations, and reduce symptoms in patients with HF with reduced ejection fraction without LVADs.^17–22^ All GDMT drug classes reduce sudden cardiac death (SCD) regardless of ICD use in the absence of LVADs.^23–25^

Current European Society of Cardiology Heart Failure 2021 guidelines recommend long-term mechanical circulatory support (MCS), i.e. centrifugal-flow pump group LVADs in selected patients when HF GDMT is insufficient or when short-term MCS has not led to clinical improvement. LVAD can both prolong life and improve the quality of life. LVADs can be utilized as bridge to transplantation or as destination therapy.^17^ Current data suggest that nearly 80% of the patients with centrifugal-flow pump group LVADs, remained alive and free of disabling stroke or reoperation at 2 years.^26^

There is very little quality data regarding the use of HF therapies in patients with LVADs. Current evidence is limited by the observational nature of the studies, relatively small sample sizes, and use of several LVAD models.^27^ Guidelines provide weak recommendations based on these data.^17,28^

While total mortality seems to be reduced with the additional HF GDMT, life-threatening arrhythmias were not specifically evaluated in clinical studies of LVAD patients. Indeed, almost two decades ago, a small single-arm, single-centre study (*n* = 15) suggested that combined therapy with chronic mechanical unloading with LVADs and HF GDMT might reverse myocardial remodelling and improve myocardial structure and function.^29–31^ A further non-randomized, multicentre study (RESTAGE-HF)^32^ suggested that HF GDMT could result in sufficient myocardial recovery to allow LVAD explantation (achieved in 40% of cases).

The role of HF GDMT continues to be uncertain.^32^ Another small (*n* = 81) non-randomized trial suggested that GDMT might have favourable effects on the structure and function beyond the beneficial effects of the unloaded myocardium attributed to LVADs.^30^

High-quality randomized, multicentre trials with appropriate endpoints are necessary to establish the role of HF GDMT in patients with LVADs.

**Table of advice.** HF GDMT

May be appropriate TO DO	Evidence	Strength
Continue GDMT titration for HF in addition to the LVAD support^29–31^	OBS	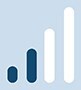

### AAD therapy

Due to the lack of data in the LVAD cohort, recommendations for medical therapy are based on data from non-LVAD HF patients, in whom amiodarone is apart from BB the only AAD not contraindicated. It remains unclear, whether it is feasible to use other AADs that have contraindications in HF patients in the LVAD cohort. Efficacy and safety of AADs in the severe HF patient cohort need to be taken into consideration when managing LVAD patients.

AADs besides BB may be used in LVAD patients for recurrent or persistent arrhythmias that lead to symptoms, reduce functional parameters or produce haemodynamic instability. In these circumstances, AADs may be used as a therapeutic option besides catheter ablation. Whether AADs should be preferred over catheter ablation remains unclear as no comparative studies in this cohort are available.

### Atrial arrhythmias

In comparison to the available data on VAs and SCD, there is a relative scarcity of information regarding the occurrence and impact of atrial arrhythmias in patients with LVADs.

Interestingly, with the current LVADs which carry a lower risk of haemocompatibility-related events, AF does not appear to be associated with increased thromboembolic or bleeding outcomes.^33^ On the contrary, AF in LVAD patients was associated with mortality, right ventricle deterioration, HF hospitalization and lower quality of life scores including 6-min walk distance, and peak VO2 consumption.^34^ In addition, AF was found to be associated with the risk of post-implantation VAs.^5^

Rhythm control strategies are not yet thoroughly examined, although the use of BB and digoxin may prove beneficial for rate control. In a study of 418 LVAD patients, AF/atrial flutter was not associated with increased mortality, thromboembolism, or bleeding, and rhythm control measures were not associated with improved outcomes.^35^ In a different study of 81 LVAD patients with AF, there was also no difference between rhythm and rate control strategy.^36^ Thus, whether the haemodynamic benefit of sinus rhythm maintenance outweighs the potential side effects of AADs used for rhythm control is controversial. There is inconclusive data concerning the role of peri-implantation left atrial appendage occlusion.^37–39^

### Ventricular Arrhythmias

Effective management of VAs in these patients necessitates collaborative efforts between electrophysiologists and HF specialists. The primary treatment options after ruling out reversible causes include adjusting LVAD settings, medical therapy, ICD implantation/programming optimization, and ablation. In terms of medical treatment, the initiation and titration of BB are advised, given their association with a reduced frequency of VA in the post-operative setting.^15^ For patients experiencing haemodynamically significant VT that does not respond to LVAD adjustments and BBs, additional medical treatment with AAD is warranted. Options include mainly amiodarone, but other drugs as sotalol, mexiletine, and intravenous sodium channel-blocking agents like lidocaine and procainamide might be advised on an individual basis.^15^

### Amiodarone use in post-LVAD arrhythmias

The most common AAD used in LVAD patients is amiodarone, which can be indicated for the treatment of atrial as well as VAs. Up to 40% of patients undergoing LVAD implant and up to 36% of patients after LVAD implantation receive amiodarone—mostly for rhythm control of AF.^10,40,41^

In patients on amiodarone treatment before LVAD implant, but not for those in whom amiodarone is initiated after LVAD implant, a higher mortality has been documented (32.9% in the amiodarone group compared with 29.6% in those not on amiodarone; *P* = 0.008).^10^ Whether amiodarone initiated before LVAD implant should be stopped after LVAD implant is unclear as data are limited. In one study, discontinuation of amiodarone after LVAD implantation led to increased recurrence of arrhythmias, but not increased mortality.^41^

Early short-term prophylactic use of amiodarone after LVAD implantation may be beneficial but evidence to support this strategy is not available. Initiation of amiodarone after LVAD implantation was not associated with a lower incidence of VA but reduced atrial arrhythmias during follow-up.^40^ Long-term amiodarone use is associated with a high risk for medication-induced complications. Of note, cases of hyperthyroid-induced hypercoagulability and pump thrombosis were published^42^ and thus, long-term prophylactic prescription of amiodarone in all patients is not advised.

**Table of advice.** AAD therapy

Advice	Evidence	Strength
**Advice TO DO**		
Amiodarone on the top BB therapy for acute and/or chronic suppression of atrial and VAs that are either symptomatic and/or lead to RV failure provided reversible cause have been excluded.^7,13,15^	OBS	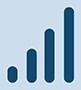
**Advice NOT TO DO**		
Long-term prophylactic use of amiodarone in all LVAD patients is not advised and may be associated with increased mortality.^10,40,41^	OBS	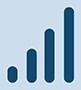

## Catheter ablation in LVAD recipients

### Catheter ablation of atrial arrhythmias

In two recent randomized controlled trials, catheter ablation of AF has been shown to improve outcomes of patients with HF with reduced ejection fraction, including those with end-stage HF eligible for heart transplantation.^43,44^ Whether these positive findings can by any means be translated to the population of patients with LVAD remains uncertain. In fact, the unique dynamics of LVAD support, where the left atrium and left ventricle are unloaded, create a distinct scenario in which the relative benefit of maintaining sinus rhythm on patient haemodynamics, thromboembolic risk, and overall outcomes is yet to be elucidated.^35^ To date, catheter ablation of AF in LVAD carriers has only been case-reported.^45,46^ The procedure was successfully performed using radiofrequency in two highly symptomatic patients without complications. Subsequent follow-up assessments revealed maintenance of sinus rhythm, contributing to patient stabilization. Additionally, a case series reported on eight patients with LVAD support undergoing ablation for cavotricuspid-dependent atrial flutter which had led to right HF. All procedures were successful and restoration of sinus rhythm resulted in symptom relief.^47^ Until more data is available, and considering the potential risk of intracardiac shunting^48^ when transseptal approach to the left atrium is required, it seems reasonable that catheter ablation of atrial arrhythmias, particularly AF, should be reserved for highly symptomatic patients only when all attempts to rhythm control with anti-arrhythmic drugs have failed.^48^ When rhythm control is not considered appropriate and rapid ventricular response is refractory to medical management, ablation of atrioventricular node and pacemaker implantation may be performed.

**Table of advice.** Catheter ablation of atrial arrhythmias

Advice	Evidence	Strength
**Advice TO DO**		
Catheter ablation of highly symptomatic cavotricuspid-dependent atrial flutter.^47^	OBS	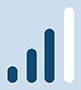
AV node ablation in symptomatic patients with AF and uncontrolled rapid ventricular response refractory to rate control medication (who are not candidates for or in whom rate control has not been successful).	OPN	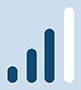
**May be Appropriate TO DO**		
Catheter ablation of highly symptomatic AF/atrial tachycardia after failure of rhythm control with anti-arrhythmic drugs.^45,46^	OBS	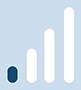

### Catheter ablation of VAs prior to LVAD implantation

Patients with recurrent VAs and advanced HF might be candidates for both LVAD implantation and catheter ablation.^6,49^ The optimal sequence of treatment, whether ablation should be performed prior to or after LVAD implantation, is not known and multiple factors should be considered in the decision-making.

On the one hand, in some cases, VA ablation may stabilize the patient and postpone or even prevent the need for LVAD implantation. Additionally, a history of VAs before LVAD is a major predictor of VAs after implantation,^3,5,50–56^ and ablation may potentially decrease VA burden after LVAD that could otherwise complicate the early post-implantation period. This might be also applicable to some patients with no history of VAs that are at very high risk.^2,3^ Finally, pre-implantation ablation might be reasonable in case where the VA will not be accessible after LVAD implant (i.e. originating from epicardium) and an electrophysiologic study in highly selected patients may help to elucidate the presence of such a substrate.

On the contrary, LVAD candidates are often fragile patients with end-stage HF, severely depressed LV ejection fraction, prone to peri-procedural acute haemodynamic deterioration, requiring intravenous vasopressors and inotropic agents to support cardiac output during the procedure, or even temporary mechanical support, although its benefit in terms of outcomes in uncertain.^57,58^ An individual risk assessment is necessary to decide whether mechanical support is necessary during VT ablation for a given patient. Thus, if an ablation is considered, an accurate pre-procedural risk stratification is essential to minimize the risk of peri-procedural complications^59^ and every effort should be made to optimize the haemodynamic status before the procedure. Finally, up to 9–24% of VTs after LVAD implantation are due to the apical scarring from the LVAD cannula,^60–62^ and one may keep in mind that VAs may still occur despite a successful pre-LVAD ablation.

**Table of advice.** Catheter ablation of VAs prior to LVAD implantation

Advice	Evidence	Strength
**Advice TO DO**		
Optimization of the haemodynamic status and careful assessment of potential risks and benefits of catheter ablation of VAs in LVAD candidates.	OPN	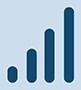
**May be Appropriate TO DO**		
An ablation procedure prior to LVAD implantation in patients with a history of recurrent VAs to reduce the arrhythmia burden.	OPN	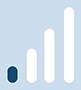
An electrophysiology study prior to LVAD implantation in selected patients with suspected epicardial substrate to guide peri-implantation surgical VA ablation.	OPN	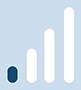

### Peri-implantation surgical VAs ablation

During LVAD implantation, the epicardium and some endocardium (through the ventriculotomy for the inflow cannula) are exposed for mapping and peri-implantation surgical VA ablation. This is important as access to the epicardial space for catheter ablation is limited after LVAD implantation due to adhesions and the location of the LVAD pump itself. Although subsequent surgical epicardial ablation via limited thoracotomy has been reported,^63^ peri-implantation surgical ablation was proposed for patients with a history of recurrent VA^64–69^ with a presumed epicardial substrate (*Table 1*).

**Table 1:** Published case series on peri-implantation VA ablation

	*N*	Access	Type of mapping	Ablation	Recurrence during follow-up (%)
Emaminia *et al.*^64^	2	Both epi/endo	Pre-operative EP mapping	Cryo	0
Mulloy *et al.*^65^	7	Both epi/endo	Pre-operative EP mapping	Cryo	28
Patel *et al.*^66^	5	Both epi/endo	Intra-operative EnSite	4-mm tip + Cryo	40
Moss *et al.*^67^	36 mapped 2 ablated	Epi only	Intra-operative EnSite	Cryo	50
Kunkel *et al.*^68^	2	Epi only	Intra-operative EnSite	Cryo	100
Tankut *et al.*^69^	10	Both epi/endo	Pre-operative ECG and imaging	4-mm tip + Cryo	50

Surgical ablation can be guided by pre-operative imaging, electrophysiologic study and intra-operative mapping. The latter may be challenging due to limited epicardial access in patients after previous coronary bypass surgery, the necessity of selective lung ventilation for accessing the epicardial left lateral aspect of the heart, low reliability of precordial leads during surgery, limited accessibility of endocardial mapping, and electromagnetic interference (EMI) limiting the use of some electro-anatomical mapping systems.^66^ Although conventional irrigated tip catheters were used for ablation, surgical radiofrequency, or cryoablation are more appropriate for creation of lesions. Despite positive results of small series,^65^ cases of pump thrombosis after endocardial cryoablation have been reported^70^ raising concern about thromboembolic risk associated with perioperative surgical ablation. Currently, an ongoing prospective trial (PIVATAL, NCT05034432)^71^ randomizes patients with a history of previous VA to either peri-implantation VA ablation or conventional medical therapy with the primary endpoint being the total VA episodes.

**Table of advice.** Peri-implantation surgical VAs ablation

May be appropriate TO DO	Evidence	Strength
Peri-implantation surgical VA ablation targeting in selected patients with recurrent VAs that failed AAD and/or previous ablation, specifically, if arrhythmia substrate is not accessible by endocardial approach (e.g. confined to epicardium).^65,66^	OBS	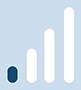

### Catheter ablation of VAs after LVAD implantation

**Figure 2:**
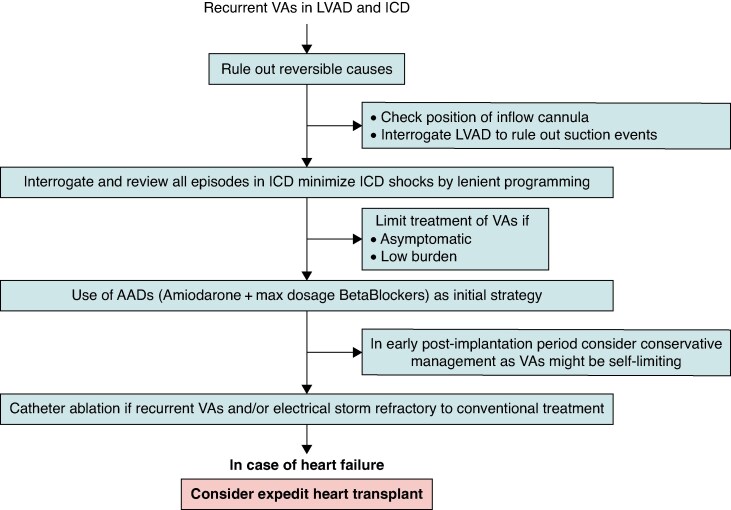
Management strategies for LVAD patients with recurrent VAs and ICD.

In patients presenting with recurrent VA episodes after LVAD implantation, a secondary cause for VA should be always ruled out (*Figure 2*). Non-sustained VT could be related to canula irritation or suction, which is even more likely when haemolysis is documented, VA depends on respiration or is linked to cough. Transthoracic or transoesophageal echocardiography (TOE) and LVAD interrogation should be used to diagnose these phenomena. If present, a decrease in the LVAD’s rounds per minute and/or optimization of LV volume may stop the VT.

Indication of invasive treatment should consider the timing of VAs. Early VTs during LVAD post-implantation period are common, especially, when occurring on high inotropic and vasopressor support.^72^ Due to the self-limiting nature of these early VAs, treatment with AADs is usually sufficient.

For patients without any symptoms and preserved RV function, VAs might be well-tolerated and no treatment might be necessary. In patients likely to undergo early heart transplant, a more conservative approach may be taken. On the other hand, in patients with anticipation for ‘long bridge’ or destination LVAD therapy, the risk of VA recurrences is higher and catheter ablation may be a reasonable option.

For recurrent symptomatic VTs, which do not respond to AAD therapy and/or ICD reprogramming, catheter ablation should be proposed after discussion between the electrophysiology and LVAD teams. Retrospective case series showed the feasibility of VT ablation with different LVAD devices.^62,73^ When pooling the different studies, non-inducibility of the clinical VT was achieved in 78% and peri-procedural complications occurred in 9% (no death). Major complications (5.5%) included two groin pseudoaneurysms requiring surgery, two cerebrovascular events, and one cardiogenic shock.

Ablation allowed VT storm termination in 90% of patients. After a mean follow-up of 9 ± 9 months, 56% did not have VT recurrence with significant VT reduction in the remaining patients, 23% underwent cardiac transplant, and 48% died. The place of pulsed field ablation remains to be determined in this indication. For patients with VAs recurring despite catheter ablation, the use of stereotactic radiotherapy^74^ or stellate ganglion block^75^ has been proposed. However, reported clinical experience for stereotactic radiotherapy is limited to five patients with a positive impact in three of them. Three patients underwent a heart transplant shortly after irradiation (one for worsening HF in two and because of arrhythmia recurrence). No device failures were reported.

**Table of advice.** Catheter ablation of VAs after LVAD implantation

Advice	Evidence	Strength
**Advice TO DO**		
Mechanical causes of VAs, such as cannula irritation, should be ruled out. These events can be diagnosed by echocardiography, LVAD interrogation or evidence of haemolysis.	OPN	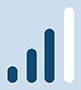
Catheter ablation for recurrent symptomatic VAs that do not respond to AAD therapy and/or ICD reprogramming.^1,2,5,6^	OBS	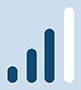
**May be appropriate TO DO**		
In the presence of recurrent VA, ICD reprogramming with a long detection time and favouring ATP over shocks may be useful	OPN	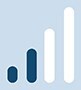

### Management of ES—the role of drug therapy and catheter ablation

ES is a life-threatening condition characterized by repetitive episodes of sustained VAs (VT or fibrillation) over a short period of time.^7,76,77^ ES was reported in 1 out of 10 patients during the first months after LVAD implantation, often occurring during the initial 30 post-operative days.^6,49,78,79^ The short-term mortality after ES is high, and one-third of patients may die within 15 days post-ES.^49^

LVAD patients often haemodynamically tolerate VAs, even ventricular fibrillation, and may therefore receive numerous painful electrical shocks while being fully conscious. Efforts should be made to avoid such a situation. In patients without LVAD support, the management of ES is a multidisciplinary multi-step approach based on a comprehensive diagnostic and clinical assessment, an escalation of medical therapy, and catheter ablation if indicated.^77^ A similar approach may be suggested for LVAD patients, as described in the recent EHRA/HRS/LAHRS clinical consensus statement on ES.^80^ First, acute triggers, which are often present during the post-operative period (i.e. adrenergic stimuli, inotropic drugs, electrolyte imbalance, ischaemia, or QT prolongation), should be identified and treated. ICDs should be reprogrammed to avoid appropriate but unnecessary shocks (longer detection rates, less aggressive ATP therapies, or disabling ICD shocks if the patient is monitored).^80^ Anti-arrhythmic therapy, based on amiodarone, mexiletine, and/or BBs (preferentially non-selective) are advised unless contraindicated.^7,76,77^ IV magnesium, associated with supplementation of potassium should be preferred for patients with Torsades de Pointes. Mild to moderate sedation can be started to reduce sympathetic tone. If VAs are refractory to medical therapy, deep sedation^81^ or neuromodulation.^82^ Stellate ganglion block may be particularly attractive in LVAD patients since it may be performed at the bedside in unstable patients, and is less invasive than other neuromodulation alternatives.^75^ Successful radiofrequency ablation of VAs has been shown to improve survival in patients with ES and no LVAD. This procedure can also be performed in LVAD patients^60,62,83^ with a good safety/efficacy profile. In a recent series about ES in LVAD patients, only 10–14% benefited from catheter ablation at the acute stage.^49,78^

**Table of advice.** Management of ES

Advice TO DO	Evidence	Strength
A multidisciplinary management including various actors (electrophysiologist, HF specialist, anaesthesiologist, cardiac surgeons, nurses) is necessary for LVAD patients presenting an ES	OBS	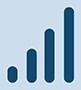
Identification and treatment of acute triggers (like adrenergic stimuli, inotropic drugs, electrolyte imbalance, ischaemia, or QT prolongation) that are often present during the post-operative period	OPN	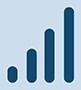
The usual therapeutic strategies used for ES (including cardioversion/defibrillation shocks or antitachycardia pacing, AADs, adrenergic blockade, sedation/anxiolysis, and pharmacological haemodynamic support) are appropriate in patients with LVAD.^49,78^	OBS	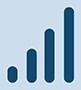
Catheter ablation of VAs is adviced in selected LVAD patients with refractory ES presumed it is performed in high volume expert centre.^60,62,83^	OBS	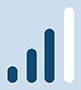

## Procedural aspects of catheter ablation in LVAD patients

### Pre- and peri-procedural imaging

**Figure 3:**
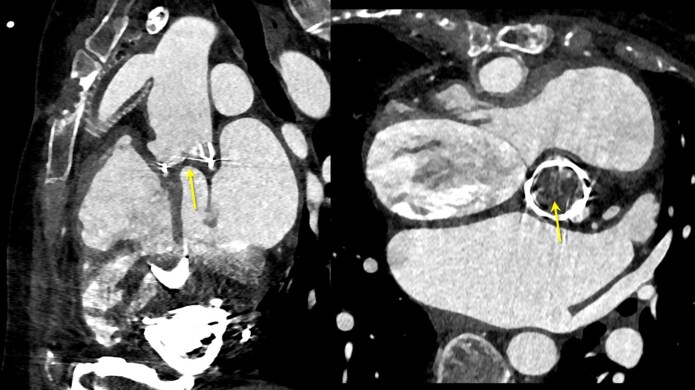
An example of an intra-aortic thrombus on a CT scan (arrow).

**Figure 4:**
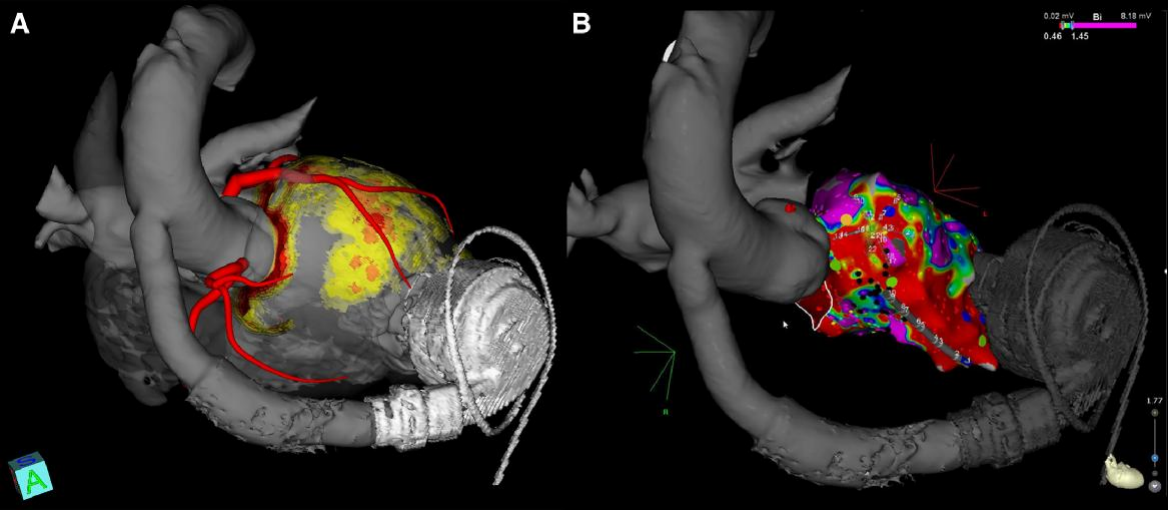
(A) CT scan of a patient with HeartMate 3 and ischaemic cardiomyopathy treated with dedicated software to be integrated into a 3D mapping system. (B) Integration of the images in the 3D mapping system with the bipolar voltage map.

**Figure 5:**
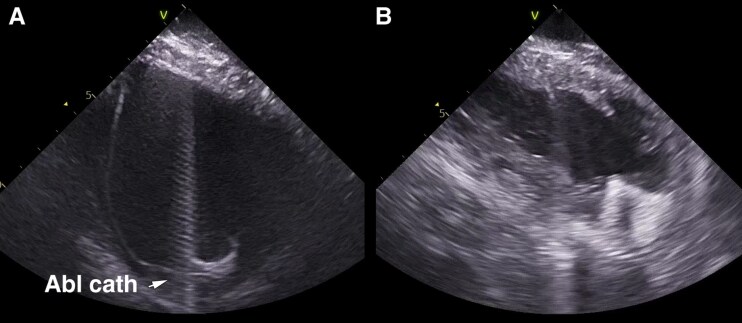
ICE during VT ablation in an LVAD patient. (A) Normal-sized LV cavity with the ablation catheter close cannula. (B) Small ‘decompressed’ LV cavity due to unloading by LVAD.

Because of the absence or limited transaortic flow, a thrombus may develop in the aortic root (*Figure 3*). Therefore, pre-procedural imaging is crucial to rule out intracardiac thrombus as for any VT ablation,^84^ but also to rule out intra-aortic thrombus, particularly when retrograde access is planned. This can be performed by echocardiography or cardiac CT scan. A CT scan may also help to identify cannula position and the arrhythmogenic substrate, especially in ischaemic cardiomyopathy. CT scan protocol has been described in detail previously.^84^ These images can be merged into a 3D mapping system to help navigation^60,73^ (*Figure 4*). Cardiac magnetic resonance imaging (MRI) is contraindicated given the incompatibility due to the highly ferromagnetic components.^85^ Intracardiac echocardiography (ICE) may be helpful to rule out intra-aortic or intra-cardiac thrombus,^86^ but is performed only after the beginning of the procedure. ICE can be also used for guidance of transseptal puncture, catheter navigation, monitoring of LV filling during VT, visualization of cardiac anatomy, and position of inflow cannula (*Figure 5*).

**Table of advice.** Pre- and peri-procedural imaging

Advice	Evidence	Strength
**Advice TO DO**		
Pre-ablation echocardiography and/or CT imaging is advised to rule out intra-aortic and/or intracardiac thrombus	OPN	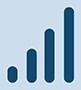
**May be appropriate TO DO**		
Pre-ablation CT for visualization of individual anatomy, the position of inflow cannula, and identification of arrhythmogenic substrate^1,2^	OBS	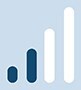
ICE to rule out the presence of intracardiac/aortic thrombus and for the guidance of transseptal puncture, catheter navigation, monitoring of LV filling during VT, visualization of individual anatomy and position of inflow cannula^2,3^	OBS	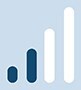
**Advice NOT TO DO**		
MRI In patients With LVAD^85^	OPN	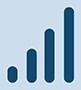

### Signal filtering and the role of ECG in the location of the VT exit site

Analysis of the ECG during VT permits a rapid localization of the exit site. This information can be used before the ablation procedure to minimize mapping time and guide ablation. Multiple ECG algorithms can be used,^87^ however, in LVAD patients their value might be limited. In the series of Anderson et al.,^62^ the presumed exit site of VT based on 12-lead ECG did not correspond to the ablation site in 45% of cases. This might be explained by progressive diffuse myopathy, anatomical distortion from LVAD placement, and LV decompression. On the other hand, non-invasive electrocardiographic mapping^88^ was shown in a case report to be effective for the identification of the exit site of VT in an LVAD patient.

**Figure 6:**
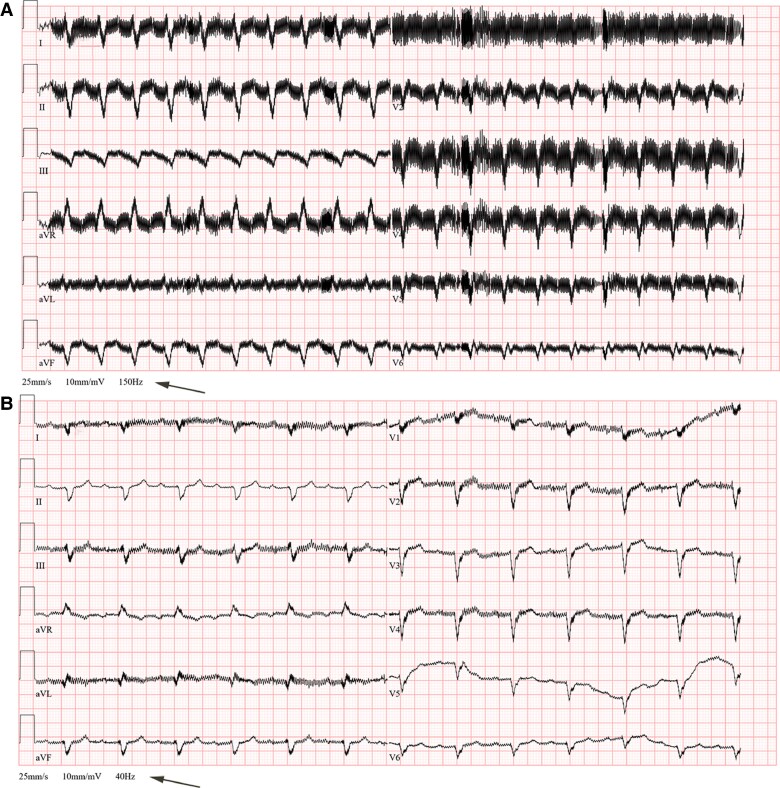
An example of 12-lead ECGs recorded in a patient with HeartMate3 using conventional filter setting (A) and after adjusting the low-pass filter to 40 Hz (B).

All LVADs generate EMI, which manifests as high-frequency noise artefacts on the surface 12-lead ECG.^73^ Reducing the low-pass filter to 40 Hz (*Figure 6*) or adding a bandstop filter can exclude signals responsible for this artefact and improve the clarity of the ECG.^89^

**Table of advice:** Signal filtering and the role of ECG in the location of the VT exit site

Advice TO DO	Evidence	Strength
Adjusting the low-pass filter and adding a bandstop filter to reduce the artefacts to improve waveform tracings and quality of the ECGs.^73,89^	OBS	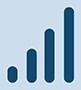

### EMI and selection of mapping system

Continuous-flow LVADs generate electrical artefacts on 12-lead ECGs but also are responsible for EMI. LVADs may interfere with some of the 3D mapping systems that establish a low-energy magnetic field around the patient's chest to detect catheter positions and navigation during ablation.^90^ EMI is dependent on the distance to the turbine, the pump speed, and the type of the mapping catheter and may result in inaccurate catheter localization, electro-anatomical point acquisition, inadequate respiratory compensation, vector orientation, and contact force readings.^60^ It typically occurs when the catheter is in close proximity to the apical inflow cannula (reported already at 8 cm).^91^ Although EMI rarely completely prohibits mapping, several measures were proposed to reduce EMI: (1) placement of mapping reference patches far from the inflow cannula (lower on the patient's back)^62^; (2) reduction of LVAD pump speed (in cooperation with the HF team)^62,91^ since slower speed is associated with less EMI and allows the visualization of the catheter at shorter distance from LVAD. (3) The use of a non-magnetic cardiac system in the solely electrical impedance mapping mode can be done to minimize the risk of EMI.^92^

**Table of advice.** EMI and selection of mapping system

May be Appropriate TO DO	Evidence	Strength
Placement of mapping reference patches far from the inflow cannula (lower on the patient's back)	OPN	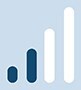
Reduction of LVAD pump speed in cooperation with the HF team to minimize EMI.	OPN	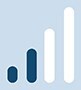
Use of a non-magnetic mapping system in the solely electrical impedance mode to minimize EMI.	OPN	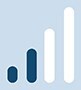

### Mapping strategies and ablation targets

During VT ablation procedures, haemodynamic support with LVAD prevents haemodynamic deterioration and facilitates mapping of otherwise haemodynamically unstable VT.

The induction of VTs is advisable as they are tolerated in the majority of patients. This allows use of activation and entrainment as the pre-dominant mapping strategies. Left ventricular electro-anatomical mapping can be performed in 75% of cases, using activation mapping and entrainment (60%), substrate mapping (20%), a combination of these (16%), or pace mapping in a few cases.^62^ Scar-related reentry is observed in 90% of cases,^62^ arrhythmias originating from the inflow cannula are present in only a minority,^60,62,93,94^ and focal, micro reentry, bundle branch reentry VT, or PVC–triggered arrhythmias in a few.^62,93^

Expected challenges with mapping were rare without relevant impact on the procedural efficacy. While the use of ICE has been suggested to assess LV filling during VT, and assist catheter position and contact, it is not used by all operators.^62^ Although sometimes utilized without issue,^94^ it is unknown if multi-spline catheters can be safely used in LVAD patients.

The primary goal of the procedure should be aimed at elimination of the clinical VT. Given the extensive arrhythmogenic substrate in most LVAD patients, it is unclear, whether ablation targeted at complete elimination of all inducible VTs is always achievable and would not lead to an excessive increase of procedure-related complications.

**Table of advice.** Mapping strategies and ablation targets

Advice TO DO	Evidence	Strength
During VT ablation procedures, mechanical support with LVAD can prevent haemodynamic deterioration and facilitate activation mapping of otherwise haemodynamically unstable VA.	OPN	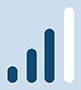
The induction of VT is advisable because tolerated in the majority of patients, allowing activation and entrainment as the pre-dominant mapping strategy.	OBS	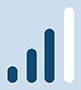

### Complications of catheter ablation in LVAD patients

In experienced centres, the incidence and nature of complications of VT ablation in patients with implanted LVAD devices are comparable to or slightly higher than in other structural heart disease non-LVAD patients. A meta-analysis comprising 110 patients found an overall incidence of complications of 9.4% with major complications occurring in 5.5% of cases (*Table 2*).^62^ Most complications are comparable to those identified in non-LVAD VT ablation cases and relate to groin access site or thromboembolic events. Increased risk of bleeding complications in LVAD patients may occur from continued need for uninterrupted anticoagulation during the procedure. LVAD-related specific complications include management and manipulations to the cannulas (catheter entrapment, pump thrombosis) or transseptal access (post-procedure persistent atrial shunting). While to the best of our knowledge no cases with catheter entrapment in the inflow cannula have been reported, caution is needed. One comparative analysis revealed VT ablation to be an independent predictor of pump thrombosis^95^ and independent of the peri-procedural anticoagulation regimen. The risk of pump thrombosis depends on the specifications of the LVAD and increases immediately after VT ablation, may remain increased for several weeks, and may relate to cerebrovascular events but the exact incidence remains unclear. Specific caution when mapping and ablating around the inflow cannula is advised as this appears to be associated with the highest incidences of pump thrombosis.^61,95^

**Table 2:** Overview of complications associated with catheter ablation in LVAD recipients

	Complication	Incidence	Reference
Minor	Groin haematoma	3.6–4.4% (4/110)	Anderson *et al*.^62^
Major	Cerebrovascular events	1.8% (2/110)	Anderson *et al*.^62^
Major	Groin/surgical repair	1.8% (2/110)	Anderson *et al*.^62^
Major	Cardiogenic shock	0.9% (1/110)	Anderson *et al*.^62^
Major	Pump thrombosis	Rare to 11%	Grinstein *et al*.^95^ Anderson *et al*.^62^
Major	Persistent atrial septal defect and right-to-left shunt	Rare	Oates *et al*.^48^ Tamura *et al*.^96^ Wang *et al*.^97^
Major	Catheter entrapment in percutaneous VAD cannula	Rare, not reported yet for LVAD	D’Angelo *et al*.^98^

Rare cases^48,96,97^ of persistent atrial septal defect with right-to-left shunting after transseptal access with iatrogenic hypoxaemia have been reported. TOE imaging may help to detect and percutaneous atrial septal occlusion may help to prevent consequences.

### Vascular cannulation and access to cardiac chambers

Safe vascular cannulation and access to the left atrium or ventricle are important to reduce complications and morbidity from ablation procedures. Procedures should ideally be performed under continued uninterrupted anticoagulation therapy (see Section ‘Peri-procedural anticoagulation management’). Therefore, vascular access site complications are of most concern. Ultrasound-guided vascular access has been shown to reduce major and overall groin complications in patients undergoing catheter ablation.^99^ Therefore, ultrasound guidance for venous and/or arterial access is advised.^60–62,95,100–102^ This is especially important as in continuous-flow LVAD, arterial pulse may not be palpable in the groin.

In VT ablation cases, usually at least one venous (pacing catheter for induction, transseptal access sheath) and one arterial access (LV retrograde access, invasive blood pressure measurement—also via radial artery possible) is performed. In AF ablation cases at least one transseptal access is required.

For VT ablation, LV access is usually needed and can be gained either via transseptal puncture or retrograde aortic approach (*Table 3*). The transseptal access appears to be the preferred route in 60–76% of cases and both, transseptal and retrograde in 1.1–56%, somewhat depending on the preference of the operator.^60–62,95^ If epicardial access is needed after LVAD implantation surgical access and limited blunt dissection may be feasible but data and cases are limited.^100^ Pre-ablation imaging may help to determine the ablation target site and guide decision on the preferred access route.

**Table 3:** Pros and cons of different access routes used for LV ablation

Access route	Pro	Con	To be contemplated
Transseptal	No passage through the aorta and aortic valve No arterial groin puncture needed No passage of LVAD outlet	Remaining persistent atrial shunt	Rule out left atrial appendage thrombus if AF Not if mechanical mitral valve prosthesis Ultrasound-guided venous puncture
Transaortic/retrograde	No need for transseptal puncture Invasive arterial pressure monitoring	Arterial groin puncture with increased risk of haematoma Large arterial sheath Risk of iatrogenic aortic dissection/regurgitation	Rule out aortic thrombus Not if mechanical aortic valve prosthesis Ultrasound-guided arterial groin puncture Aortic valve opening required

If a transaortic approach to the LV is performed, temporary reduction of pump speed may be helpful to allow sufficient opening of the aortic valve. Aortic root thrombosis can be detected in up to 10% of LVAD patients.^103^ Therefore, some centres routinely perform TOE or CT imaging to rule out aortic thrombosis.^48,103,104^ In LVAD patients with AF, thrombus in the left atrial appendage should be excluded using TOE or CT. Mechanical damage to the aortic valve caused by catheter manipulation may increase the aortic insufficiency with adverse haemodynamic consequences. On the other hand, rare cases of right-to-left shunting after transseptal access due to LVAD suction have been reported (see complications).^48,105^

**Table of advice.** Vascular cannulation and access to cardiac chambers

Advice	Evidence	Strength
**Advice TO DO**		
Use of ultrasound guidance for venous and/or arterial groin puncture in all LVAD patients to reduce access site complications.	OPN	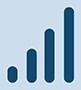

### Peri-procedural anticoagulation management

Due to a complex interplay of factors including haemolysis, platelet activation, platelet adhesion, and inflammation, patients with the current generation of continuous-flow LVADs have an increased risk of both bleeding and thrombotic complications.^106^ In fact, pump thrombosis and stroke contribute significantly to morbidity and mortality in LVAD carriers. This mandates LVAD patients to be on chronic oral anticoagulation with vitamin K antagonists targeting an international normalized ratio (INR) goal between 2.0 and 3.0.^107^ Recently, the addition of aspirin to VKA was shown to be redundant and even harmful.^108^ Considering the additional thromboembolic risk associated with endocardial ablation, and acknowledging reported cases of pump thrombosis after VT ablation close to the inflow cannula, it seems reasonable to perform the intervention under uninterrupted therapeutic anticoagulation, with the administration of heparin to maintain an ACT ≥ 300 s throughout the procedure and avoiding reversal of anticoagulation with protamine after ablation.^61,95,107,109^ In addition, although specific data on this scenario is lacking, administration of unfractionated heparin or low molecular weight heparin in the presence of a sub-therapeutic INR after ablation would be advisable until therapeutic INR levels are reached.^107^

**Table of advice.** Peri-procedural anticoagulation management

Advice	Evidence	Strength
**Advice TO DO**		
Performing catheter ablation under uninterrupted oral anticoagulation.^60^	OBS	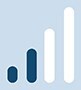
Administration of heparin for the ablation procedure to achieve and maintain an ACT ≥300 s.	OPN	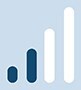
Peri-procedural administration of unfractionated heparin or low molecular weight heparin in the presence of a sub-therapeutic INR until a therapeutic INR is reached (2–3).	OPN	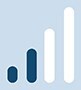
**Advice NOT TO DO**		
Reversal of anticoagulation with protamine after ablation should be avoided.	OPN	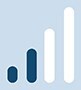

### Peri-procedural haemodynamic monitoring

Peri-procedural haemodynamic monitoring plays an important role during catheter ablation of VT in LVAD patients. The automatic sphygmomanometer might not measure the blood pressure adequately in patients with continuous-flow LVAD, and an arterial line for continuous blood pressure monitoring is typically needed.^15^ Careful and close monitoring of vital signs, fluid balance, arterial blood gas analysis, and parameters of peripheral perfusion (including lactate levels) is also important during the procedure, particularly if procedure duration is not short.^110^ Additionally, LVAD flow and power should be closely monitored during catheter ablation.^15^ Direct recording of the central venous pressure measured by an internal jugular central venous catheter could also be useful.^59^ In selected high-risk patients, even if VA may be tolerated due to LV support, it might lead to RV failure and elevation of right-sided pressures: thus, intra-procedural invasive monitoring with a pulmonary artery catheter could be used in such cases, especially when the catheter has already been positioned before the procedure in the intensive care unit. However, direct left atrial pressure recording or left ventricular end-diastolic pressure recording have been described as alternatives to pulmonary artery catheter monitoring.^59^ Non-invasive cerebral oximetry is also useful to evaluate cerebral desaturation during the procedure.^59,110^

Beyond intra-procedural monitoring, post-procedural close monitoring in the intensive care unit is usually needed after catheter ablation of VT in LVAD patients.

**Table of advice.** Peri-procedural haemodynamic monitoring

Advice	Evidence	Strength
**Advice TO DO**		
Careful peri-procedural monitoring (including invasive arterial pressure monitoring, LVAD flow and power monitoring, vital signs and perfusion assessment, and cerebral oximetry) during catheter ablation of VT in patients with continuous-flow LVAD.	OPN	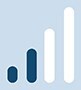
**May be Appropriate TO DO**		
Venous pressure, invasive monitoring with a pulmonary artery catheter or direct left atrial pressure recording or left ventricular end-diastolic pressure recording in selected patients	OPN	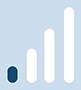

## Gaps in knowledge

There is very little quality data regarding the use of devices, ablations, and even implementation of guideline-directed HF medical therapies in patients with LVADs. Current evidence is limited by the observational nature of the studies, relatively small sample sizes, and the use of several LVAD models. In addition, the decision in LVAD patients relies heavily on very individual circumstances and on personalized risk and benefit analysis that has to be performed in every single patient.

We believe that based on the current developments in mapping and catheter ablation treatments in cardiac arrhythmias, dedicated trials should be made regarding its clinical effectiveness in both atrial and VAs pre- and post-LVAD implantation.

The vast majority of the clinical advice in this document is based on extrapolations derived from our current knowledge and practice in patients with HF in general rather than based on clinical randomized studies in LVAD patients. Thus, many key issues are still unclear:

- What is the impact of GDMT in reducing VAs in LVAD patients?
- What is the optimal anticoagulation management for LVAD patients in general and specifically for CIEDs implantation and ablation?
- In LVAD patients with AF, does the haemodynamic benefit of sinus rhythm maintenance outweigh the potential side effects of AADs used for rhythm control?
- Does catheter ablation of AF improve symptoms and outcomes in patients with LVAD support?
- The role of intra-procedural or post-procedural left atrial appendage procedures for the prevention of thromboembolic events is to be established.
- Who are the appropriate candidates and what should be the optimal mapping and ablation strategy for peri-implantation surgical VA ablation?
- Should catheter ablation be systematically performed for recurrent VAs and/or ES in LVAD recipients and what should be the optimal timing?
- Whether the VT-LVAD risk score would help identify, which patients would benefit from catheter ablation is unknown.
- Can pre-LVAD catheter ablation of VAs decrease the incidence of post-LVAD VAs and ES?
- Does prophylactic anti-arrhythmic treatment during the early period-at-risk (30 days post-LVAD implantation) decrease the risk of ES?
- Considering the severity of the underlying disease and the amount of inducible VAs in some LVAD patients, what should be the optimal endpoint of the catheter ablation procedure?
- Should peri-procedural antibiotic prophylaxis be administered routinely for catheter ablation in LVAD patients?
- Is the usage of multi-electrodes catheters during mapping in LVAD patients safe?
- What might be the clinically relevant interactions between electrophysiology technology and LVAD machinery?
- The utility of stellate ganglion blockade in the management of ES in patients with LVAD appears to be promising but needs further study.

## Conclusions

Both atrial and ventricular tachyarrhythmias occur frequently in LVAD patients. The potential treatment strategies include mainly medical therapy, anti-arrhythmic drugs, optimization of programming in patients with implanted ICDs and catheter ablation in those with significant arrhythmias resistant to medical treatment. For proper management, close cooperation of HF specialists, electrophysiology team, and also cardiac surgeons is required before, during and after the LVAD implantation procedure. There is a need for further accumulation of data and properly conducted studies in this population.

## Data Availability

There are no new data associated with this article.

## Reviewers

- **Luigi Di Biase (Review coordinator)** MD, PhD, FACC, FESC, FHRS: Montefiore Health System at Albert Einstein College of Medicine, New York, NY, USA
- **Isabel Deisenhofer:** Department of Electrophysiology, German Heart Center Munich, Technical University of Munich, Munich, Germany
- **Alessio Gasperetti** MD PhD: School of Medicine, Johns Hopkins University, Baltimore, MD, USA
- **Mélèze Hocini:** Hôpital cardiologique du Haut Lévêque, Cardiology—Cardiac Electrophysiology Department, Pessac, France and IHU Liryc—L’Institut des maladies du rythme cardiaque, Site Hôpital Xavier Arnozan, Pessac, France
- **Francisco Moscoso Costa:** Department of Cardiology, Hospital de Santa Cruz, Carnaxide, Lisbon, Portugal
- **Brenda Moura:** Armed Forces Hospital, Porto, Portugal and Faculty of medicine of the University of Porto, Portugal
- **Hadi Skouri:** School of Medicine, Balamand University, Beirut, Lebanon and Sheikh Shakhbout Medical City, Abu Dhabi, UAE
- **Carlo Gabriele Tocchetti** MD, PhD: Director, Internal Medicine Unit for cancer patients, Department of Translational Medical Sciences (DISMET), Center for Basic and Clinical Immunology Research (CISI), Interdepartmental Center for Clinical and Translational Research (CIRCET), Interdepartmental Hypertension Research Center (CIRIAPA), Federico II University, Via Pansini 5, Edificio 2, 80131 Naples, NA, Italy
- **Maurizio Volterrani:** Professor of Exercise Science and Medicine, San Raffaele Open University of Rome, Director of the Cardiopulmonary Department, IRCCS San Raffaele Roma, 00166 Roma, Italy
- **Reza Wakili:** Deputy Director, Department of Cardiology and Vascular Medicine, Head of Arrhythmia Service and Electrophysiology
  University Hospital Frankfurt, Goethe-University Frankfurt, Theodor-Stern-Kai 7, 60590 Frankfurt am Main, Germany
  ZIM—Med. Klinik 3—Kardiologie, Angiologie
