# Correction to: Drug therapy and catheter ablation for management of arrhythmias in continuous flow left ventricular assist device’s patients: a Clinical Consensus Statement of the European Heart Rhythm Association and the Heart Failure Association of the ESC

**DOI:** 10.1093/europace/euaf086

**Published:** 2025-05-22

This is a correction to: Petr Peichl, Antoni Bayes-Genis, Thomas Deneke, Ovidiu Chioncel, Marta deRiva, Maria Generosa Crespo-Leiro, Antonio Frontera, Finn Gustafsson, Raphaël P Martins, Matteo Pagnesi, Philippe Maury, Mark C Petrie, Frederic Sacher, Offer Amir, Drug therapy and catheter ablation for management of arrhythmias in continuous flow left ventricular assist device’s patients: a Clinical Consensus Statement of the European Heart Rhythm Association and the Heart Failure Association of the ESC, *EP Europace*, Volume 26, Issue 11, November 2024, euae272, https://doi.org/10.1093/europace/euae272

The following changes have been made to the originally published manuscript. The names and affiliations for the reviewers have been removed from the author list and replaced within a separate section in the body of the article.

